# Comparative mechanical testing for different orthodontic aligner materials over time - in vitro study

**DOI:** 10.4317/jced.59569

**Published:** 2022-06-01

**Authors:** Clara-Marie Nowak, Ahmed Othman, Dragan-Alexander Ströbele, Constantin von See

**Affiliations:** 1 Researcher in the digital technologies in dentistry and CAD/CAM department- Danube Private University- Austria; 2 Assistant Professor, Research Center for Digital Technologies in Dentistry and CAD/CAM, Department of Dentistry, Faculty of Medicine and Dentistry, Danube Private University, 3500 Krems, Austria; 3 Professor and Director of Research Center for Digital Technologies in Dentistry and CAD/CAM, Department of Dentistry, Faculty of Medicine and Dentistry, Danube Private University, 3500 Krems, Austria

## Abstract

**Background:**

The purpose of the present study is to mechanically evaluate and compare the forces over 12 hours on different orthodontic aligners manufactured by Polyethylene terephthalate glycol (PETG).

**Material and Methods:**

Twelve orthodontic aligner specimens will be produced by a thermoforming laboratory vacuum machine. All specimens will be divided into two equal groups, group A representing Duran (Scheu Dental GmbH, Iserlohn, Germany) and group B representing Erkodur (Erkodent, Pfalzgrafenweiler, Germany). These specimens will be fabricated via CAD/CAM technology by scanning a Frasaco model (Henry Schein Dental, Gallin, Germany) using D 800 (3Shape, Copenhagen, Denmark) and printed via a Varseo S machine using Varseo ModelWax material (BEGO, Bremen, Germany). Group A specimens are manufactured by a Twinster thermoforming machine (Scheu Dental GmbH, Iserlohn, Germany) while group B is produced using Erkoform thermoforming machine (Erkodent, Pfalzgrafenweiler, Germany). Afterwards, a tooth will be removed from the printed model and replaced by an ivory tooth (Henry Schein Dental, Gallin, Germany) to apply forces at a predicted measured centre of resistance. The universal testing machine Z010 (ZwickRoell, Ulm, Germany) will be used for mechanical testing with 0.3 mm displacement over 12 hours. Statistical analysis was performed using Sigmaplot 13.0 (Systat Software GmbH, Erkrath, Germany). Behaviours over time were analysed using R2-regression analysis (SPSS 26.0, IBM SPSS Statistics, Armonk, USA).

**Results:**

There is no statistically significant difference in the maximum force between both groups (*p*=0.071). The mechanical testing over 12 hours showed cubic properties.

**Conclusions:**

The PETG material has no influence on the produced mechanical forces regardless of the manufacturing company. The forces over time showed no tendency towards a lower boundary of force.

** Key words:**Mechanical testing, CAD/CAM, orthodontics, thermoplastic aligner materials.

## Introduction

As a matter of fact, the recent high demand of orthodontic treatment seeking by adults has increased the clinical concern for more aesthetic, clear and comfortable appliances compared to conventional fixed appliances (1). Correspondingly, the necessity of better dental aesthetics has been embraced with clear aligner therapy, ceramic brackets, and lingual orthodontics (2). A study was conducted by Chen-Lu Liu, who found that aligners are more accepted by adult patients than conventional brackets (3). Additionally, clear aligners are highly requested within orthodontic patients (4).

In the same way, the clear aligner therapy is involved with treating various orthodontic problems, as well as the concept of fixed appliances, however with mechanical limitations. In view of the different production methods of clear thermoforming material, it gets engaged to the full teeth surfaces for orthodontic mechanical treatment to a wide range of malocclusion (5). Most of the conventional aligner companies do not require the usage of dental practitioner intervention at any stage of the treatment. However, the bonding of some attachments or buttons for controlled mechanical movements is sometimes mandatory.

Further, the aligner therapy includes a sequential usage of various transparent trays made from thermoplastic materials (6). As suitable examples of the latter, polyvinyl chloride, polyurethane (PU), polyethylene terephthalate (PET) and polyethylene terephthalate glycol copolyester (PET-G) can be stated (7). In this regard, a study was conducted by Jeong-Hyun Ryu to evaluate the different materials used for clear aligner therapy and their influence on the mechanical behaviour (8). However, the major impact on mechanical properties of materials used for clear aligners and the influence of the variations between different production companies or the tray thickness have not been yet investigated (9). Therefore, to derive the tooth movement, forces exerted, and material thickness must be considered, as these influence the extent of deflection after loading and thus have the greatest influence on force and tension (10).

In this study, two groups of thermoplastic trays made of Polyethylene terephthalate glycol (PETG) from Duran (Scheu Dental GmbH, Iserlohn, Germany) and Erkodur (Erkodent, Pfalzgrafenweiler, Germany) are mechanically tested and statistically compared. All in all, it is known that different materials cause different forces within the aligners (11). In contrast, currently it is unknown how the force applied impacts the aligners over time. Thus, we conducted a systematic *in vitro* study investigating the force-time behaviour of different aligner materials.

In orthodontic treatment methods, there are differences between fixed and removable appliances. With fixed multiband appliances, the force is effective for 24 hours (12). On the contrary, the standard wearing time for removable appliances is 12 hours (13). Since it is no longer possible to control how often the aligners are removed and for how long, it is indispensable to differ from the standard wearing time. Assuming that aligners are removed when eating and for cleaning (14). After brushing your teeth overnight, aligners are not removed, causing the assumption of a maximum wearing time of 12 hours. Aligners are removed and thus manipulate the mechanical properties (15). Overall, in orthodontics, a constant application of force is ideal. Nevertheless, this is not the case with aligners, regardless of the material, because the force cannot be kept constant and decreases over time (16). Under these circumstances, it is necessary to test the aligners for 12 hours in this *in vitro* study to investigate the force-time behaviour.

## Material and Methods

Twelve orthodontic aligners were laboratory produced by a thermoforming vacuum machine using a 3D printed Frasaco model. All specimens were divided into two equal groups, Duran A (Scheu Dental, Iserlohn, Germany) (n=6) was produced by Twinster (Scheu Dental GmbH, Iserlohn, Germany) and Erkodur B (Erkodent, Pfalzgrafenweiler, Germany) was produced by Erkodent (Pfalzgrafenweiler, Germany) (n=6). The properties of each material and its thermoforming conditions (thicknesses, temperatures, heating times and cooling times) are shown in Table 1.

**Table 1:**
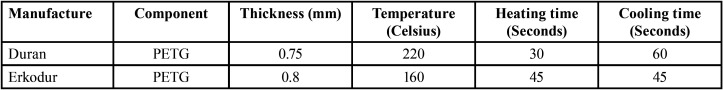
The properties of each material and its thermoforming conditions.

As shown by Elkholy F. *et al*. (17) the thermoforming process resulted in different mechanical behaviour for these materials. Two materials with similar thicknesses were selected for this study to allow a better comparison. Group A’s material thickness is 0.75 mm, whereas group B has a thickness of 0.8 mm to ensure comparable results in comparison to thicker aligner materials.

The conventional Frasaco model (Henry Schein Dental, Gallin, Germany) was 3D scanned using the model scanner D 800 (3Shape, Copenhagen, Denmark). Afterwards, the virtual Frasaco model was 3D printed using Varseo ModelWax (BEGO, Bremen, Germany) on the Varseo S 3D printer (BEGO, Bremen, Germany). After scanning the model, a block was designed using Autodesk Netfabb 2020.0 (San Rafael, USA) to ensure a fixed duplicaTable position of the printed model to the Zwick machine (Ulm, Germany) during mechanical testing. The model was cleaned for 3 minutes by placing it in an unheated, reusable ultrasonic ethanol vessel with a concentration of 96%. Accordingly, the model was removed from the ethanol bath and dried with compressed air as specified in the BEGO instructions. Henceforth, the cleaning process was repeated in a new ethanol bath with a concentration of 96% for 2 minutes and dried with compressed air. Afterwards, the tooth 21 was laboratory removed and replaced by a Frasaco tooth with an average length (22 mm), clinical crown height (12 mm), and width (8 mm) (Henry Schein Dental, Gallin, Germany). All test specimens were marked with a sample number, cleaned with compressed air, and checked for any defects before usage.

**Figure 1:**
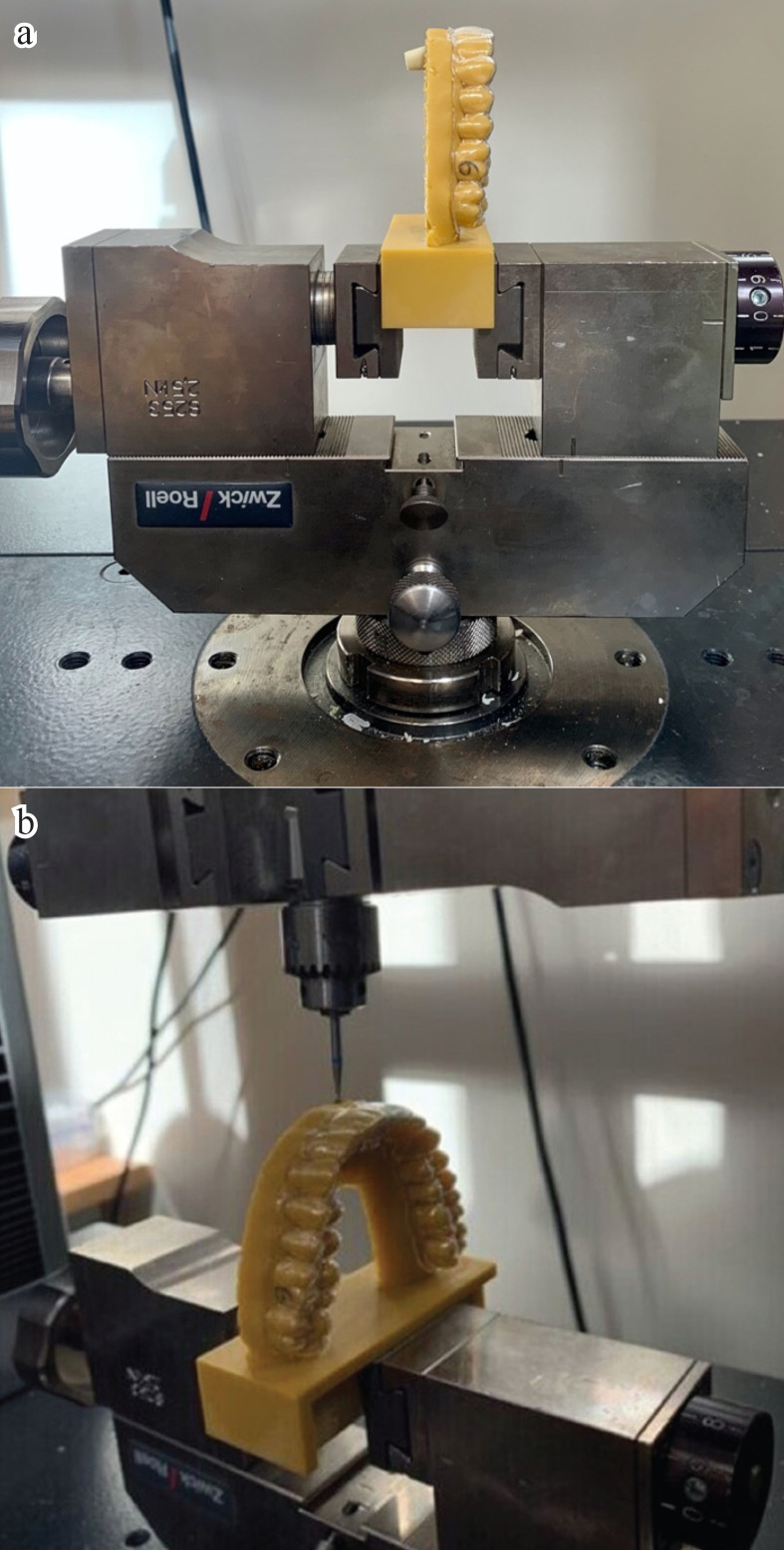
a) Modified printed Frasaco model clamped in the universal testing machine Z010. b) A constant load on the tooth.

The loading force point was manually predicted before mechanical testing and marked on the tooth by placing the centre of resistance (CR). In fact, the position of the CR is dependent of the ratio between the crown and the root of the tooth. Hence, it is defined approximately 2/3 of the root length measured from the root’s apex to the tooth crown (18). In the final analysis, the modified model was clamped in the universal testing machine Z010 (ZwickRoell, Ulm, Germany) for 12 hours. All test specimens were exactly positioned on a clamping holding device with the same clamping pressure using a torque wrench (Fig. 1a). A constant load of 0.3 mm was applied by the machine on the tooth over a period of 12 hours (Fig. 1b). To record the data, the software TestXpert II (Zwick/Roell, Ulm, Germany) was launched on the testing machine Z010. All results were documented in Newtons.

-Statistical analysis

As a matter of fact, all data are expressed as means ± standard deviations. Granted that the data for each material and thickness were analysed using the two-tailed-P-value-test, which was used to compare the groups. With this intention the statistical analysis was performed using Sigmaplot 13.0 (Systat Software GmbH, Erkrath, Germany). Owing to the behaviours over time were analysed using R2-regression analysis (SPSS 26.0, IBM SPSS Statistics, Armonk, USA).

## Results

In a nutshell, there is no significant difference in the maximum forces applied on the two thermoforming aligners of the distinct companies (*p*=0.071). A two-tailed-P-value-test was applied to determine the results.

**Figure 2:**
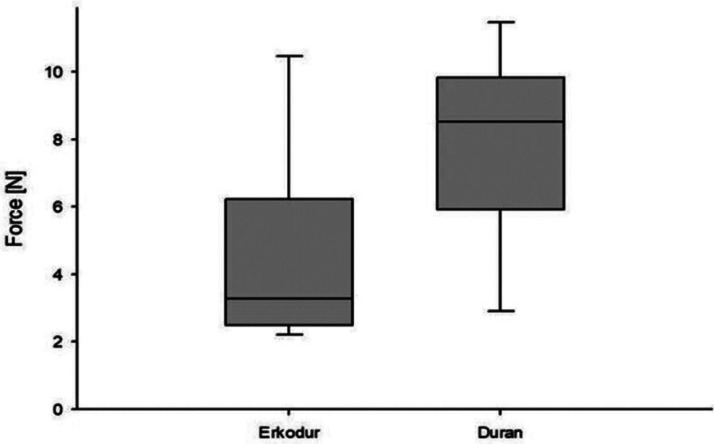
Maximum forces exerted on Erkodur and Duran.

When evaluating the results, group A (0.75 mm) and group B (0.8 mm) barely differ from each other. Hence the highest measured values were found in group A with 11.41 N, while group B performed with 10.46 N. In essence, the lowest measurements were found in group A with 2.89 N and in group B with 2.19 N (Fig. 2).

On balance, the standard deviation for group B is 3.090 and for group A is 2.918. All test results are summarized in Fig. 2. The statistical analyse was performed using Sigmaplot 13.0 (Systat Software GmbH, Erkrath, Germany).

**Figure 3:**
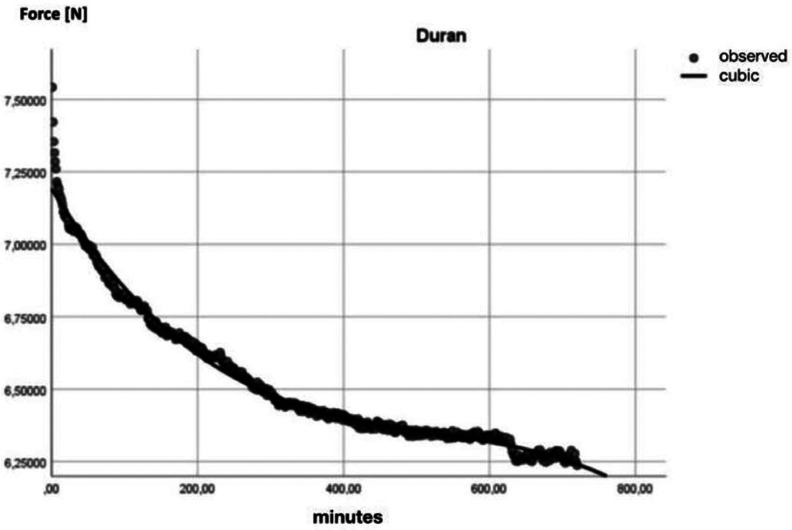
Regression analysis Duran displayed on a graph.

**Figure 4:**
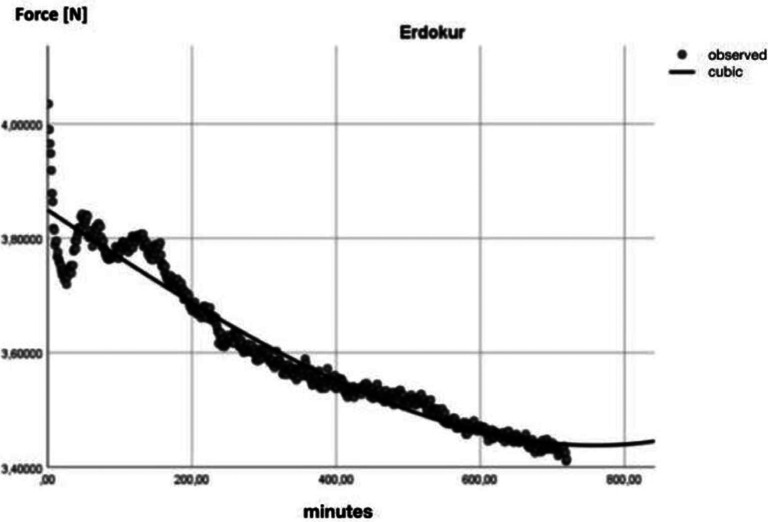
Regression analysis Erkodur displayed on a graph.

Finally, a R2-regression analysis (SPSS 26.0, IBM SPSS Statistics, Armonk, USA) was executed on the data of each group, showing the cubic behaviour of the forces over time on each manufacturer ( Table 2,  Table 3, Figs. 3,4).

**Table 2:**
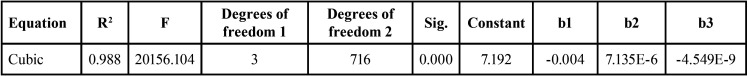
Regression analysis Duran.

**Table 3:**
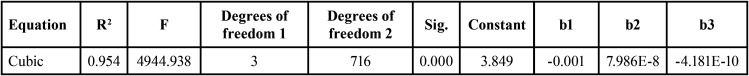
Regression analysis of Erkodur.

## Discussion

In recent years, the demand for orthodontic treatment using the aligner therapy has rapidly increased due to better perceived aesthetics and its consideration as a comfortable alternative to fixed orthodontic devices (19). Nevertheless, to assess the mechanical evaluation and comparison of different aligner orthodontic companies using the Polyethylene-terephthalate glycol material (PETG), it is necessary to acknowledge the results of the *in vitro* study of Elkholy F. *et al*. (20). However, no standard methods exist for evaluating the mechanical properties of thermoplastic materials used for the fabrication of aligners. Thus, the present study aims to establish a comparative method to evaluate aligner.

According to the current literature, it elucidates that the force applied on orthodontic aligners are mainly depended on the material thickness, whereas the manufacturing company is not as relevant (21). Furthermore, it is mandatory to be aware of a multimodal system in this *in vitro* study. Hence, in this investigation it is crucial to differ between, on one hand, the measurement of the force layer and, on the other hand, a 3-dimensional measurement in 6 axes. Including the randomly choice of the tooth 21, it is essential to consider that the incisors usually have adjacent teeth within a dental arch, which has an influence on the movement during the orthodontic aligner therapy. In addition to this, it is vital to recognise that it is not significant to the material whether the tooth movement is isolated or not, but rather reasonable, so it is a systematic *in vitro* study, eventhough not fully reproducible into the clinic implementation. In order to establish a 1:1 replicable study, it is necessary to perform another study, taking the range of forces and torsional moments into account, which will be a 3-dimensional measurement in 6 axes.

As shown by Tamburrino F. *et al*., thermoforming material was evaluated to investigate the influence of different production companies on the tooth movement (22). Apparently, there was no statistically significant difference between both groups, which was proved in this study as both materials show almost the same results. Furthermore, the regression analysis over time showed a cubic behaviour, which means firstly no constant characteristics and secondly no tendency towards a lower boundary of force. In short, those two parameters are equally important and subsequently necessarily needed for the clinical orthodontic usage. Therefore, it was found that the materials used in this study showed insufficient mechanical properties for orthodontic usages. However, some factors require further research. Specially to ensure optimal clinical application, it must be noted that the tooth movement achieved in this study cannot be transferred directly if no corresponding *in vivo* values are known to date and a specification cannot be predicted.

In the literature, analyses of the physical properties of recognized thermoplastic materials can be found. Indeed, the assessment of the orthodontic force established physical necessary values and determined the factors that exert the physical forces on the materials (23). A study by Inoue *et al*. showed that the water absorption of Duran and Erkodur was significantly higher after 2 weeks than after 24 hours (24). Additionally, a significant reduction in the modulus of elasticity was observed for both materials by applying constant strain (25). In summary, for comparing the orthodontic forces exerted by aligners the literature suggests the modulus of elasticity measurement is suitable, although the test performed by Brockmeyer P. *et al*. is not identical to the application in clinical treatment (26). Previous *in vitro* studies have evaluated the colour stability of various types of clear aligners. In contrast to the current study, the aspect of colour changes of the clear aligners due to an increased consumptions of beverages such as coffee, black tea and red wine was examined and compared with a control group in which the aligners were exposed to distilled water only. As a result, the analysis of Bernard G. *et al*. showed that the different aligner materials did not show any colour changes after 12 hours, admittedly after 7 days all materials showed colour instabilities except the control group, which only absorbed water (27). Thus, given these points, it can be concluded that if a high aesthetic orthodontic treatment is important for the patient, one should avoid contact with coffee, black tea and red wine during the aligner therapy and only consume water.

In addition to this, a prospective clinical study analysed the thickness of the clear aligners and compared the thickness after the thermoforming process and after 10 days of physiological use. As a matter of fact, the clear aligners in the study of Cervinara F. *et al*. were made of polyethylene terephthalate glycol copolyester (PET-G) material with a thickness of 0.75 mm, which allows to transfer the results of this *in vivo* study to the present *in vitro* study (28). The authors of the clinical study concluded that thin materials exerted a greater force on tooth movement than thicker materials, but the thinnest standardized aligners (0.5 mm) exerted a significant overloading on the periodontium (28). Hence, the material thicknesses in the current study does not show any consequential damage to the periodontium, but still exert a high force for efficient tooth movement. In that case, it can be assumed that the aligners have similar results in clinical use as in this *in vitro* study.

However, other variances besides time for instance heat, material thickness or the removal of the aligners must be considered (29). With attention to those details, it would further falsify a clinical result, which is why further investigations are necessary to clarify how the permanent application of force in the jaw occurs *in vivo*. In general, for this reason further systematic clinical studies must be conducted. Frequently, aligners are not only worn for 12 hours, albeit over 2 weeks and changed afterwards (30). Further tests, which are reproducible for a period of 2 weeks, must be conducted to find out how the mechanical properties change, and which forces are still acting after 2 weeks and how much they deviate from the maximum forces.

## Conclusions

Overall, the results show no significant difference between Erkodur and Duran. While evaluating both firms in detail, the aligner material PET-G has an insufficient influence on the tooth movement, as its mechanical force cannot be kept constant over time. Thus, the materials used in this study showed insufficient mechanical properties for orthodontic usage.
